# The Eukaryotic-Specific ISD11 Is a Complex-Orphan Protein with Ability to Bind the Prokaryotic IscS

**DOI:** 10.1371/journal.pone.0157895

**Published:** 2016-07-18

**Authors:** Robert Yan, Martin Friemel, Claudia Aloisi, Martijn Huynen, Ian A. Taylor, Silke Leimkühler, Annalisa Pastore

**Affiliations:** 1 Maurice Wohl Institute, King’s College London, 5 Cutcombe Rd, SE5, London, United Kingdom; 2 Department of Molecular Enzymology, Institute of Biochemistry and Biology, University of Potsdam, Karl-Liebknecht-Str. 24-25, 14476 Potsdam, Germany; 3 CMBI 260, Radboud University Medical Centre, PO Box 9101, 6500 HB, Nijmegen, The Netherlands; 4 The Francis Crick Institute, Mill Hill Laboratory, The Ridgeway, London, NW7 1AA, United Kingdom; Sant Joan de Déu Children's Hospital, SPAIN

## Abstract

The eukaryotic protein Isd11 is a chaperone that binds and stabilizes the central component of the essential metabolic pathway responsible for formation of iron-sulfur clusters in mitochondria, the desulfurase Nfs1. Little is known about the exact role of Isd11. Here, we show that human Isd11 (ISD11) is a helical protein which exists in solution as an equilibrium between monomer, dimeric and tetrameric species when in the absence of human Nfs1 (NFS1). We also show that, surprisingly, recombinant ISD11 expressed in *E*. *coli* co-purifies with the bacterial orthologue of NFS1, IscS. Binding is weak but specific suggesting that, despite the absence of Isd11 sequences in bacteria, there is enough conservation between the two desulfurases to retain a similar mode of interaction. This knowledge may inform us on the conservation of the mode of binding of Isd11 to the desulfurase. We used evolutionary evidence to suggest Isd11 residues involved in the interaction.

## Introduction

The iron-sulfur cluster pathway (ISC) is responsible for the assembly of iron-sulfur clusters in proteins, for molybdenum cofactor (Moco) biosynthesis and for tRNA thiolation [1–3]. Its disruption results in a number of inherited diseases [4–7]. The central component of the pathway is Nfs1 (NFS1 in humans), a highly conserved desulfurase enzyme which catalyses the conversion of cysteine to alanine and liberates sulfur for further use in the pathway [3].

Much of what is known about the structure and enzymatic mechanism of eukaryotic Nfs1 comes from analogy with the *E*. *coli* orthologue, IscS and the *Azotobacter vinelandii* homologue NifS [8–11]. These enzymes are dependent on a pyridoxal phosphate (PLP) cofactor which forms a series of intermediate adducts with the cysteine substrate [12, 13]. The process generates a persulfide intermediate on a cysteine located on a mobile loop of the enzyme that undergoes a conformational change to facilitate the transfer of sulfide to other acceptor proteins, such as Isu1 in the case of Fe-S cluster assembly, or TusA in Moco synthesis and tRNA thiolation [14, 15]. Ferredoxin is required for providing electrons for the release of sulfide from the persulfide intermediate [16–18].

Despite the high conservation between orthologues, there are some surprising differences between the two systems. IscS is a highly active enzyme that is fully functional without the need of accessory factors. All the Nfs1 proteins tested are, on the other hand, virtually inactive by themselves and require Isd11 (ISD11 in humans), an accessory protein not found in prokaryotes, to be functional [2, 19]. Reports suggest that Isd11 helps to stabilise Nfs1 by preventing its aggregation and degradation [20, 21]. Isd11 is a member of the larger LYR complex I family, named after a highly conserved tripeptide motif close to the N-terminus [22, 23]. This motif was shown to be present in several iron-sulfur cluster biogenesis related chaperones as for example in maturation factors of complex II [24].

Proteins from the IscS and Nfs1 families also differ dramatically in their behaviour with respect to frataxin, the protein responsible for Friedreich’s ataxia [25]. Frataxin has opposite effects on the activity of these orthologues, causing inhibition of IscS and activation in Nfs1 [26–28]. This *in vitro* difference in behaviour was tracked down to the identity of the desulfurase, as both frataxin and IscU bacterial and human orthologues are otherwise exchangeable [27]. A possible explanation could be the need to have Isd11 in the assay carried out with Nfs1 or specific differences in the IscS/Nfs1 sequences which could determine different interactions with the other components. It has for instance been suggested that binding of Isd11 on Nfs1 affects the conformation of the cysteine loop of Nfs1 [19], implicating Isd11 in the transfer of sulfur. It has alternatively been suggested that Isd11 could have a profound effect on the quaternary structure of this protein by converting it from a homodimer to a trimer of Nfs1-Isd11 heterodimers [29]. A recent bioinformatics study that utilised docking of predicted structures of Nfs1 and Isd11 suggested instead binding of Isd11 on the dimerization site of Nfs1 in order to prevent oligomerization and aggregation [30].

A better understanding of the structural differences between IscS and NFS1 would clarify the difference in behaviour but the *in vitro* study of isolated Isd11 has so far been problematic because when expressed in *E*. *coli* it proved insoluble and attempts to refold significant quantities from inclusion bodies have been unsuccessful [19, 29].

In this work, we describe a method to express human ISD11 in a soluble form which allowed us to characterize it in isolation by a combination of circular dichroism (CD) and size exclusion chromatography. Sequence analysis allowed us to predict residues of the Isd11 family potentially involved in interaction with Nfs1. We show using mass spectrometry and nuclear magnetic resonance (NMR) that, surprisingly because of its absence in prokaryotes, ISD11 is able to interact with bacterial IscS. Altogether, our results indicate retention of the binding properties of Nfs1 and IscS proteins despite their evolutionary distance.

## Materials and Methods

### Sample production

Human ISD11 was cloned in a pGEX-4T (resulting in vector pZM96) vector for expression with an N-terminal GST tag with a thrombin protease site, to allow removal of the tag. Protein expression was carried out in Rosetta2 (DE3)pLysS cells. Cells were grown in LB medium to OD_600_ 0.7 and protein expression induced with 0.5 mM IPTG and cells further incubated overnight at 18°C. For NMR samples, protein was uniformly labelled with the isotope ^15^N ([U-^15^N]-ISD11) by using M9 minimal medium supplemented with ^15^N ammonium sulfate for cell growth. Cells were lysed by sonication in lysis buffer (PBS, 0.5 mM TCEP, 0.5% v/v Igepal, 1mg/mL lysozyme, 1 mg/mL DNase I). Clarified supernatant was incubated with Glutathione Sepharose 4B resin (GE Healthcare) at 4°C for 1 hour. The resin was washed with PBS and the protein eluted with elution buffer (50 mM Tris-HCl pH 8, 10 mM reduced glutathione, 0.5 mM TCEP) using the manufacturer’s guidelines. Eluted protein was cleaved with thrombin (Merck) overnight at 4°C whilst being extensively dialysed against PBS. The following day the cleaved protein was passed through Glutathione Sepharose 4B resin to separate liberated ISD11 from GST and remaining uncleaved GST-ISD11 protein. Thrombin cleavage yielded full length ISD11 protein (UniProt accession number: Q9HD34) with five additional amino acids at the N-terminus consisting of the sequence GSPEF. The protein was further purified by size exclusion chromatography (SEC) using a 16/600 Superdex75pg column in SEC buffer (20 mM Tris-HCl pH 7.5, 150 mM NaCl, 0.5 mM TCEP). Protein sample purity and identity were checked by mass spectrometry (MS) by the LRI/NIMR Proteomics and Metabolomics Core Technology Platform (Francis Crick Institute).

Recombinant *E*. *coli* IscS (UniProt accession number: P0A6B7) was expressed and purified as described previously [31]. Briefly, IscS was expressed with an N-terminal His-tag and purified by Ni-affinity. The His tag was removed using TEV protease, which leaves the additional residues GA at the N-terminus. Coexpression of untagged IscS (pSL219, [32]) and His-ISD11 (pZM6, [2]) was carried out in *E*.*coli* CL100 *ΔiscS*^*-*^ cells.

### Analytical SEC and Multi-angle laser light scattering

Analytical SEC was carried out using 100 μL of sample at concentrations ranging from 30 μM to 120 μM and either a 10/300 Superdex 200 increase or a 10/300 Superdex 75 column at a flow rate of 0.5 ml/min of SEC buffer. The molecular weight of ISD11 in solution was determined using multi-angle laser light scattering coupled with size exclusion chromatography (SEC-MALLS). Samples (100 μl) of SEC purified ISD11 (200–400 μg) were applied to a Superdex 200-INCREASE 10/300 GL column equilibrated in 50 mM sodium phosphate 100 mM NaCl at pH 6.8 and 3 mM NaN_3_ at a flow rate of 1.0 ml/min. The column was mounted on a Jasco HPLC controlled by the Chrompass software package. The Scattered light intensity of the column eluent was recorded at fifteen angles using a DAWN-HELEOS-II multi-angle laser light scattering detector (Wyatt Technology Corp., Santa Barbara, CA). Protein concentration was determined from the refractive index change (dn/dc = 0.186) using an OPTILAB T-rEX differential refractometer equipped with a Peltier temperature-regulated flow cell, maintained at 25°C (Wyatt Technology Corp., Santa Barbara, CA). The wavelength of the laser in the DAWN-HELEOS and the light source in the OPTILAB-rEX was 658 nm. The weight-averaged molecular weight of material contained in chromatographic peaks was determined using the ASTRA software version 6.1 (Wyatt Technology Corp., Santa Barbara, CA). Briefly, at 1 s intervals throughout the elution of the ISD11 peaks the scattered light intensities together with the corresponding protein concentration were used to construct Debye plots (KC/R_θ_ vs sin^2^(θ/2)). The weight-averaged molecular weight was then calculated at each point in the chromatogram from the intercept of an individual plot. An overall average molecular weight and polydispersity term for each species was calculated by combining and averaging the data from the individual measurements.

### CD analysis

Jasco-815 CD and Varian Cary 50 Bio UV-Visible spectrophotometers were used to measure CD and absorption spectra. CD measurements were carried out in 20 mM Tris-HCl, at pH 8 and 150 mM NaCl on ISD11 samples at 113 μg/mL. A wavelength scan was performed between 190 and 260 nm. Thermal denaturation curves were recorded at the fixed wavelength of 220 nm in the 10° to 90°C temperature range recorded with 0.2°C intervals. The mean residue molar ellipticity was calculated using the formula [θ]_MRW_ = θ x MRW / (10 x c x l) where MRW is the molecular weight divided by the number of residues less 1, c is the concentration of the sample in mg/ml and l is the cell path length in cm. The melting profile of ISD11 was fitted by a two-state transition model, implemented in SIGMA-Plot^®^.

### NMR experiments

NMR experiments were recorded at different concentrations of ISD11. The best quality data were recorded at a protein concentration of 34 μM. A ^15^N-NOESY-HSQC was monitored at 800 MHz with a mixing time of 450 ms. ^15^N-SOFAST-HMQC spectra were used to monitor [U-^15^N]-ISD11 upon titration with unlabelled IscS. A Bruker Avance III spectrometer at 600 MHz equipped with TCI cryoprobe was used for the measurements.

### MS analysis database searching

MS/MS was carried out with a Mascot instrument (Matrix Science, London, UK; version 2.4.0) set up to search the UniProt_KB2011_03a database (14423061 entries) assuming trypsin cleavage, a fragment ion mass tolerance of 0.80 Da and a parent ion tolerance of 5.0 PPM. Post-translational modifications were specified as variable modifications. Scaffold (version 4.3.4, Proteome Software Inc., Portland, OR) was used to validate MS/MS identifications. Peptide identifications were accepted if they could be established with > 90.0% probability by the Peptide Prophet algorithm [33], with a Scaffold delta-mass correction. Protein identifications were accepted if they could be established by the Protein Prophet algorithm [34] with >20.0% probability and contained at least 2 identified peptides. Proteins that contained similar peptides and could not be differentiated by MS/MS alone were grouped to satisfy the principle of parsimony.

#### Bioinformatics sequence analysis

Database searching was also used to identify residues specific of the Isd11 family. We used JackHMMER [35], starting with Isd11 from yeast, with five iterations and default settings on the UniProt proteins to obtain LYR family members most similar to Isd11. We subsequently filtered those with “skipredundant” from the EMBOSS package to obtain a set of sequences with maximally 90% identity. Using the alignment of this set of sequences provided by JackHHMER we generated a neighbor-joining tree, using the identity matrix for substitutions and correcting for multiple substitution in the calculation of the distances. In the tree we identified a set of 211 proteins that were all close relatives of Isd11. An independent search using the database OrthoMCL [36] showed that >95% of the proteins we identified as Isd11 orthologs were also categorized as Isd11 orthologs in that database. From the alignment a sequence logo was created with WebLogo [37], using only the positions that are present in the yeast Isd11 protein. In the same tree we identified clusters dominated by other members of the LYR family: LYRM2, LYRM3, LYRM5, LYRM7 and ACN9, in total containing 617 sequences. The combined alignment, again restricted to positions that are present in Isd11, was analyzed with sequence HARMONY [38] to identify the positions most specific to Isd11.

### Determination of L-cysteine desulfurase activity

L-cysteine desulfurase activity was quantified by the methylene blue method [39] following the protocol previously described [40]. Assay mixtures in a total volume of 0.2 ml contained 100 mM Tris-HCl, 200 mM NaCl, 2 mM DTT (pH 8.0), 2 μM of IscS (pETM11) and either without or with 8 μM ISD11 (separately purified from pZM96). We estimated the concentrations of the IscS samples using the absorbance of the PLP cofactor. To rule out any variation in PLP loading between the samples we further analysed the samples using SDS-PAGE and densitometry to estimate the concentrations of the proteins. The reactions were initiated by the addition of L-cysteine (100 μM) and incubated for 5 min at 37°C before the reactions were stopped by the addition of 20 μl 20 mM N,Ndimethyl-p-phenylenediamine in 7.2 M HCl and 20 μl 30 mM FeCl_3_ in 1.2 M HCl. Samples were centrifuged for 5 min at 16,000xg. The supernatant was transferred to 96 well plates and methylene blue was determined at 670 nm. The standard curve was recorded with sodium sulfide. The reference sample contained 100 mM Tris-HCl, 200 mM NaCl, 10 μM PLP, 1 mM DTT (pH 8.0) and 100 μM L-cysteine.

## Results

### Isolated ISD11 is polydisperse

**Fig 1:**
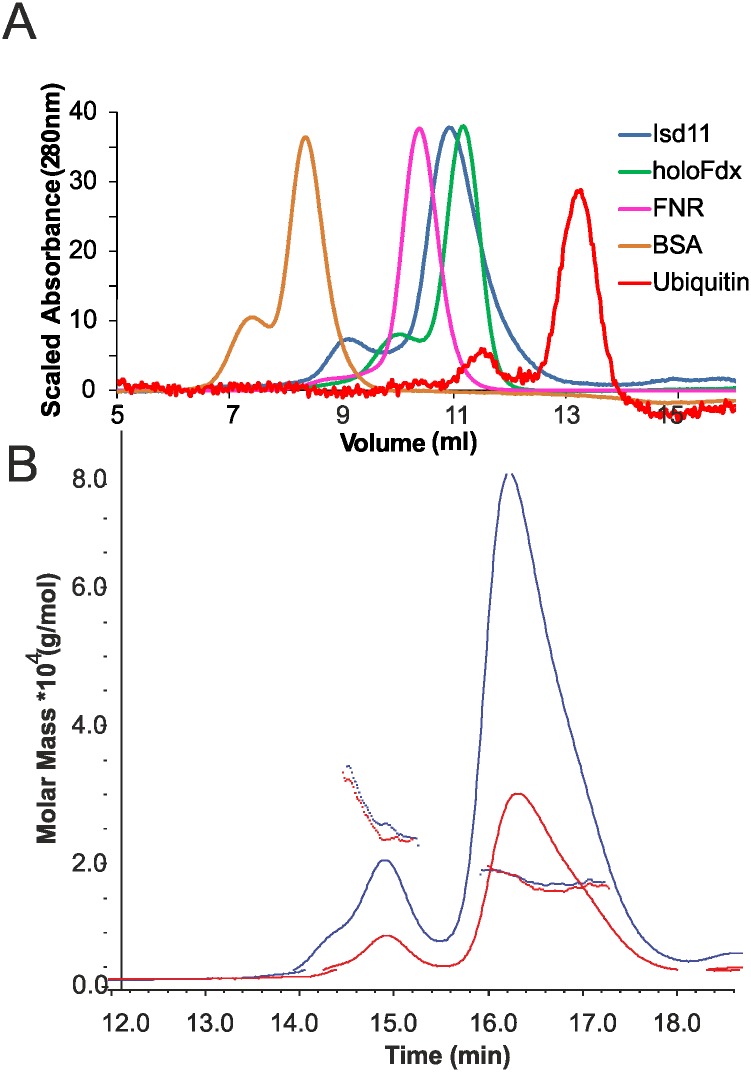
Oligomeric state of ISD11. A) Chromatograms from analytical SEC on a 10/300 Superdex 75 column for Ubiquitin, ISD11, holo-Ferredoxin (Fdx), Ferredoxin-NADP Reductase (FNR) and bovine serum albumin (BSA). B) SEC/MALLS analysis of ISD11 at concentrations of 1 mg/mL and 2 mg/mL on a 10/300 Superdex 200 increase column. Chromatograms are output from dRI detector. Points are weight-averaged molar mass calculated at 1 s intervals throughout elution of chromatographic peaks.

We established an expression system for the heterologous expression of human ISD11 in *E*. *coli*. As opposed to previous studies, we managed to obtain the protein in its soluble form by including a cleavable GST tag. After purification, we performed analytical SEC to determine the oligomeric state and compared the results to other ISC proteins (Fig 1A). ISD11 eluted in two peaks with retention volumes around 9.2 ml and 10.8 ml, implying that ISD11 exists in different oligomeric states in solution. By comparison, holo-Fdx, which is a globular monomer of ~14 kDa, eluted as a single peak with a retention volume of around 11.2 ml. This indicates that ISD11, whose molecular mass is 11.5 kDa, has a Stokes radius larger than expected for a globular monomer. To determine the oligomeric state of ISD11 more accurately, we used SEC/MALLS. Analysis of the molar masses for the species corresponding to each peak clearly indicated that ISD11 is very heterogeneous (Fig 1B). By taking an average for the molar mass over the retention time range, we estimated that the larger species were consistent with a mixture of dimer and tetramer of ISD11 and the smaller species with a mixture of monomer and dimer. However, the two peaks seem to be in equilibrium as their ratios are different with concentration and aging. In freshly purified samples from SEC, (where the concentration of the purified fraction is less than 0.5 mg/ml), the dimer is by and large the predominant species.

### Structural studies of isolated ISD11

**Fig 2:**
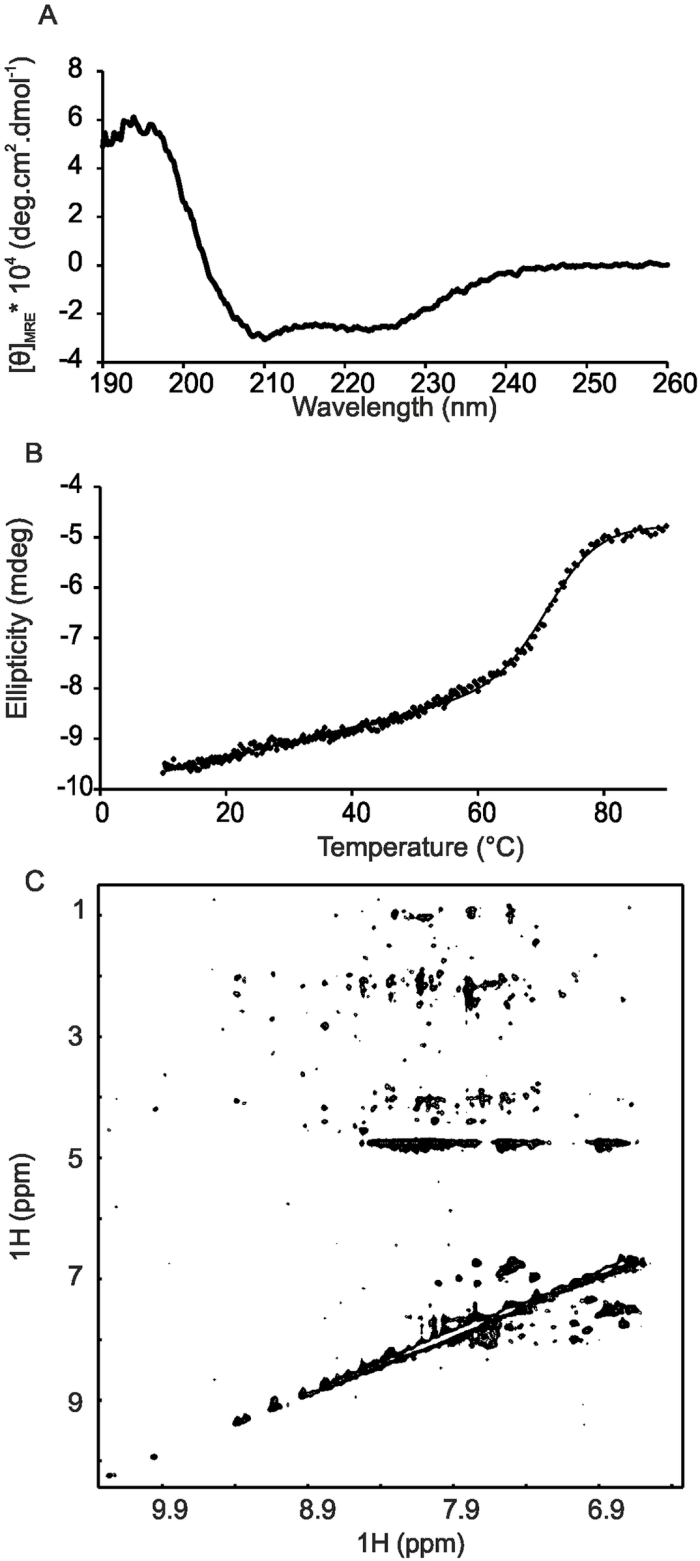
Structural characterization of ISD11. A) Far-UV CD spectrum of ISD11. B) Thermal denaturation curve of ISD11 after cleavage of the GST tag monitored at the CD signal at 220 nm. The data were fitted to a two state transition. C) Projection of a ^15^N-NOESY-HSQC spectrum of ISD11 recorded at 800 MHz with a mixing time of 450 ms. All nitrogen planes are displayed.

Analysis of the secondary structure of purified ISD11 by far-UV CD revealed a wavelength spectrum typical of α-helical proteins (Fig 2A). We estimated the secondary structure content using the K2D method [41] and found 57% helical and 10% β sheet content. This is in agreement with the secondary structure prediction and the previous ab initio modelling which both suggest a mainly helical structure [30]. The thermal denaturation curve of ISD11 revealed a high melting temperature around 67°C (Fig 2B). The value is higher as compared to the co-expressed human NFS1Δ1–55 x ISD11 complex which exhibits a melting temperature of about 61°C [7]. This could well be understood considering that in the complex, ISD11 is likely to act as a monomer and thus have a lower stability than the isolated dimer/oligomer. The stabilities in these different environments can thus easily be non-comparable. It is also not in conflict with previously reported evidence suggesting that Isd11 proteins stabilize the fold of Nfs1 since they could simply help, in a chaperone-like way, the desulfurase to fold more efficiently.

We attempted to determine the structure of ISD11 by NMR and/or X-ray crystallography. Unfortunately, we were unable to obtain any reproducible crystals (data not shown). The NMR spectrum of the protein is of low quality at concentrations amenable for NMR studies with an insufficient signal-to-noise ratio. This is consistent with the presence of polydispersity and/or conformational intermediate exchange regime. It is also compatible with the presence of higher molecular weight species in mutual equilibrium with each other. In particular, 2D NOESY spectra exhibited very few NOE crosspeaks at a variety of mixing times ranging from 40–450 ms (Fig 2C). The spectrum appreciably improved in diluted conditions but remains suboptimal for obtaining full spectral assignment. We furthermore screened sample conditions by varying pH (5.5–8.0), salt concentration (0–200 mM NaCl), using additives (50 mM Arginine/Glutamine or 4% glycerol) and temperature variation (4–40°C) but these were largely ineffective in improving the NMR spectra. This suggests that the oligomerization constants between the species in equilibrium are in the micromolar range.

### ISD11 co-purifies with IscS

**Fig 3:**
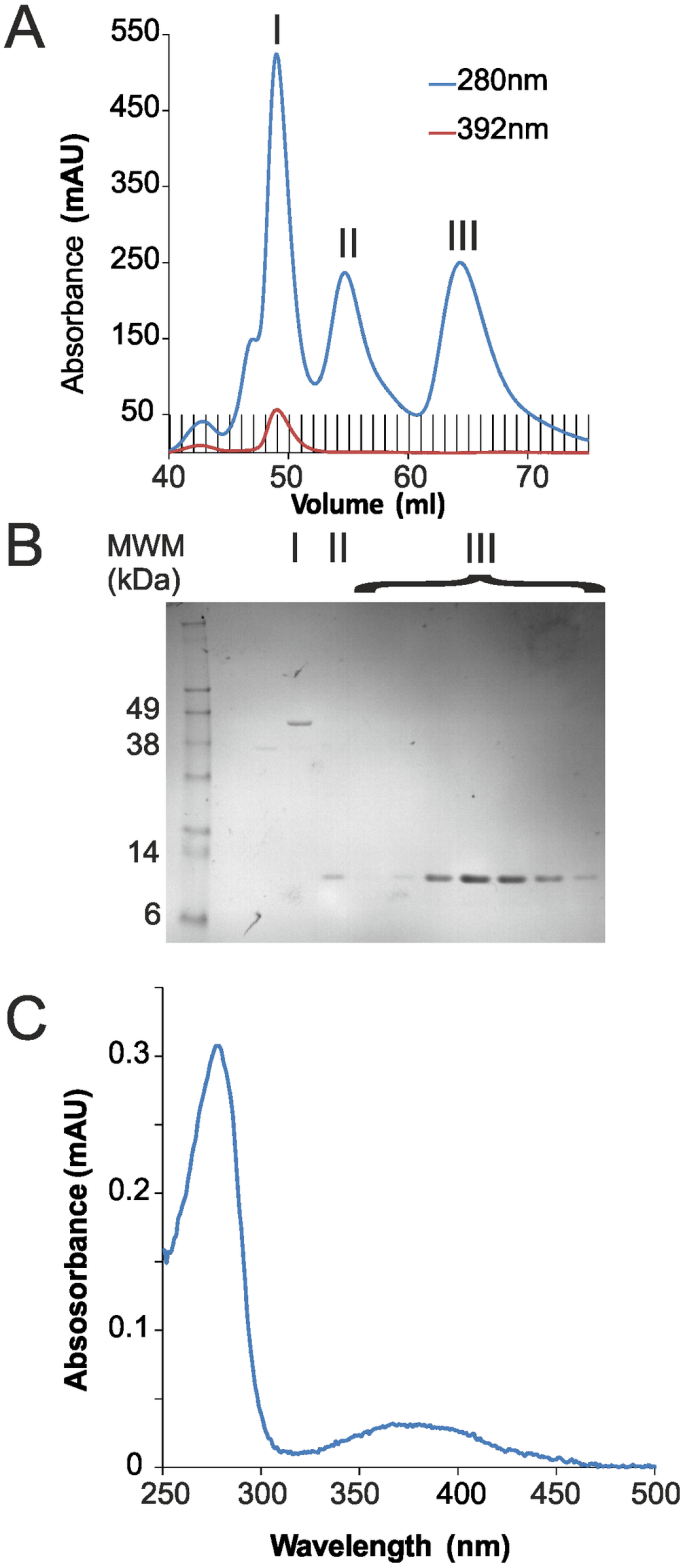
IscS co-purifies with ISD11. A) Chromatograms measured at A_280_ and A_392_ for the purification of ISD11 by SEC on a 16/600 Superdex 75 column. B) SDS-PAGE of SEC peaks I, II and III from A). C) Absorbance spectrum of co-purified peak I from A).

During purification of ISD11, we observed that a protein of ca. 45 kDa co-purified with ISD11 (Fig 3A and 3B). Analysis of the sample by MS identified the co-purified protein as the bacterial IscS. 23 unique peptides covering 64% of the IscS sequence were identified (S1 Table). A full list of identified peptides and corresponding MASCOT scores is given in the supporting information. We measured the UV/Vis spectrum of this co-purified sample after SEC and its absorption spectrum is consistent with the presence of PLP, which is the cofactor of IscS (Fig 3C).

**Fig 4:**
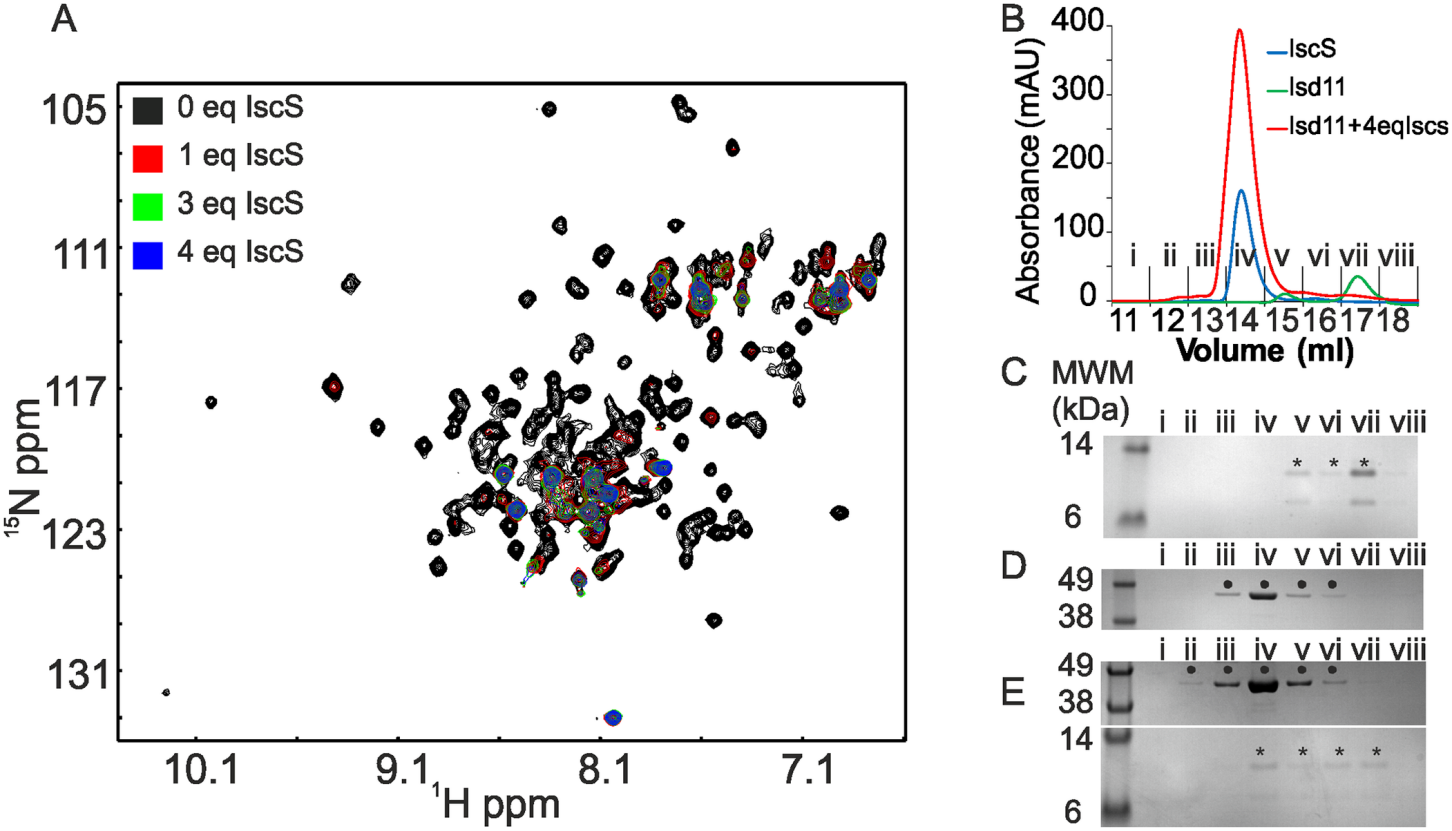
ISD11 forms a complex with IscS. A) Overlay of SOFAST-HMQC spectra of 34 μM [U-^15^N] labelled ISD11 titrated with increasing molar equivalence (eq) of unlabelled IscS. The experiment was recorded at constant ISD11 concentration. Each spectrum had to be recorded over 2h to achieve a reasonable signal-to-noise ratio. B) Analytical SEC using a 10/300 Superdex 200 increase column for the NMR sample in A) of ISD11:IscS at 1:4 molar ratio and of ISD11 and IscS separately. C), D) and E) SDS-PAGE analysis of the fractions from the SEC in (B) for ISD11 (C), IscS (D) and ISD11 titrated with 4 molar equivalents of IscS (D). Bands corresponding to ISD11 and IscS are indicated with the symbols * and • respectively.

To further characterize the interaction of ISD11 with IscS and test whether it is specific, we used NMR to monitor the ^1^H/^15^N correlation spectrum of a 30 μM sample of ISD11 upon titration with IscS. While this concentration is at the limit of structure determination or full spectrum assignment, it is sufficient to allow probing of interactions by chemical shift perturbation. Upon progressive titration of ^15^N-labelled ISD11 with unlabelled IscS, most of the peaks disappeared around 1 molar equivalents of IscS (Fig 4A). By 2–3 molar equivalents, there were no further changes of the spectrum. Only a handful of peaks remained even at 4 molar equivalence of IscS. This result is consistent with the formation of a complex between ISD11 and IscS, which would be over 90 kDa and therefore unobservable without uniform deuteration and TROSY based experiments. The peaks that remained visible are likely to correspond to regions of ISD11 remaining flexible upon interaction with IscS (e.g. the C-terminus and the amide side chains). The NMR sample was also analysed by analytical SEC to check for complex formation between ISD11 and IscS. SDS-PAGE of the fractions from the analytical SEC showed that a fraction of ISD11 co-eluted with IscS (Fig 4B). A fraction of ISD11 had also dissociated from IscS, likely because of sample dilution during SEC which would reduce the saturation of ISD11 binding sites for IscS. Nevertheless, the co-elution of ISD11 with IscS in SEC indicated that ISD11 forms a relatively tight (in the micromolar range or below) complex with IscS (Fig 4C, 4D and 4E).

The copurification of IscS (pSL219) with his-tagged ISD11 was verified via MALDI-TOF MS from an 15% acrylamide SDS-PAGE. The sequence coverage was about 46% with a corresponding MASCOT-Score of 182.

### ISD11 does not have an effect on the desulfurase activity of IscS

**Fig 5:**
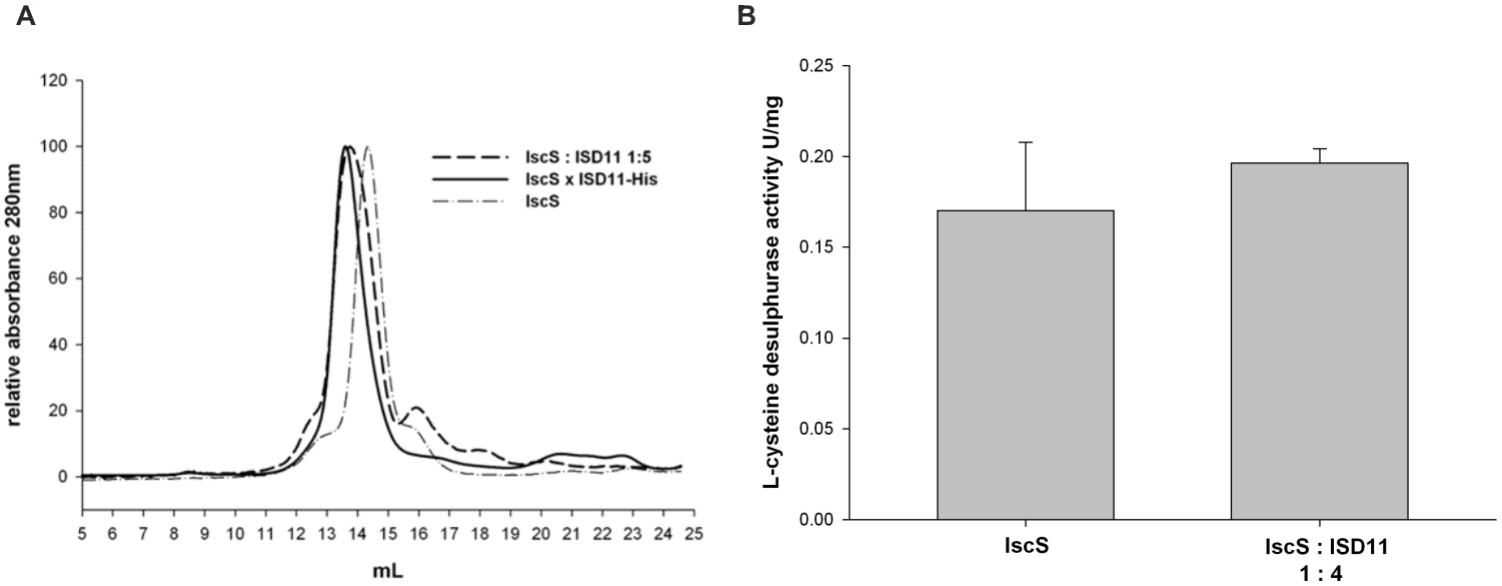
Characterization of the ISD11-IscS interaction. A) Analytical size exclusion chromatograms at 280 nm of 30 μM of either His_10_-tagged IscS, of coexpressed ISD11-His_6_ (pZM6) and IscS (pSL219), and separately purified IscS-His_10_ (30 μM) and ISD11 (150 μM, purified by GST-tag) mixed in a ratio of 1 to 5. B) L-cysteine desulfurase activity as determined by the methylene blue assay. The activity of separately purified IscS-His and IscS-His mixed with ISD11, raitio 1:4, (pZM96) was compared. The assay contained 2 mM DTT, 100 μM L-cysteine, 2 μM IscS-His and 8 μM ISD11. The assay was carried out for 5 min at 37°C. The absorbance of formed methylene blue was determined at 670 nm.

We then compared by analytical gel filtration complex formation between ISD11 and IscS as obtained from titration of the isolated proteins (His10-tagged IscS and GST-tagged ISD11 after removal of the GST-tag) with that formed by co-purification using His-tagged ISD11 (pZM6) (Fig 5A). In both cases, we observed a clear peak shift as compared to the isolated IscS that is comparable for the two samples. This suggests that a complex of the same size is formed.

To compare the *in vitro* desulfurase activity of purified IscS and assembled with ISD11, we performed the methylene blue assay using 2 μM IscS and a 1:4 IscS-ISD11 molar ratio. We observed the same L-cysteine desulfurase activity (Fig 5B), indicating that ISD11 has no effect on the activity of IscS after complex formation. This can be explained by considering that Nfs1 is highly unstable in the absence of ISD11 [20]. The presence of this protein owes to affect enzymatic activity by increasing the percentage of folded and thus active enzyme. IscS is instead stably folded anyway. Addition of ISD11 does not have any effect.

### Predicting residues involved in NFS1 interaction

**Fig 6:**
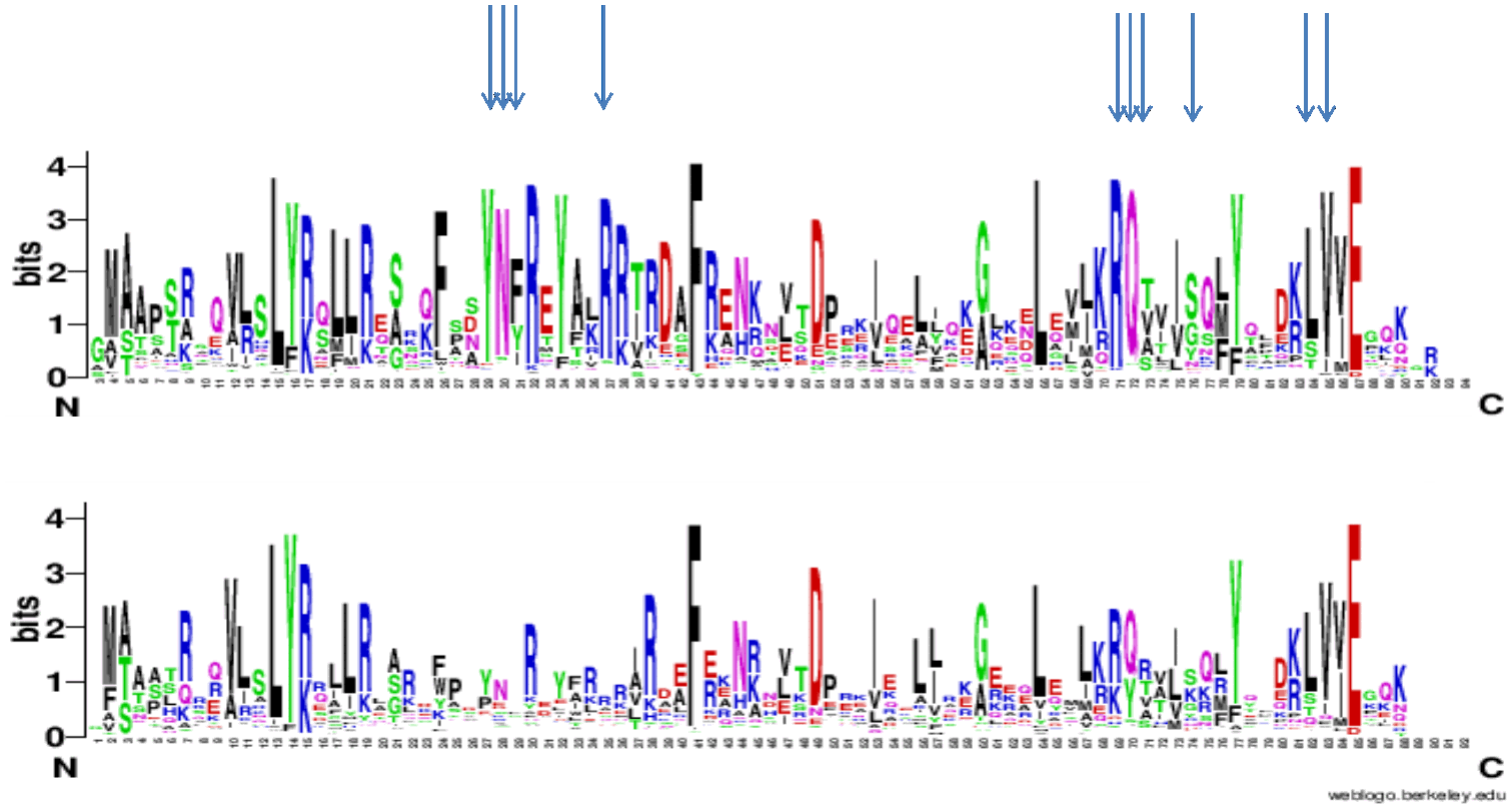
Evolutionary approach to identify the potential surface of Isd11 involved in interactions with Nfs1. Sequence logos of the Isd11 (top) and other members of the LYR families. The letter size is proportional to the degree of conservation of each residue in the multiple alignment of the family. The residues that are most specifically conserved in the Isd11 subfamily and less so in other LYR proteins are indicated with arrows. The ISD11 logo is based on an alignment of 211 Isd11 orthologues with maximally 90% identity and is limited to positions that are present in yeast Isd11. Identification of residues specific to Isd11 is based on a comparison with other members of the LYR family: LYRM2, LYRM3, LYRM5, LYRM7 and ACN9.

Finally, we set out to determine residues more specifically conserved in Isd11 rather than in the whole LYR family, hypothesizing that these would be important for the interaction with NFS1. We obtained a combined alignment of the LYR family in which we identified ISD11 orthologues and orthologues of other LYR family members that are most similar to ISD11: LYRM2, LYRM3, LYRM5, LYRM7 and ACN9. Subsequently, we used the tool sequence HARMONY [38] to identify the residues specifically conserved in the Isd11 subfamily and containing other amino acids in the other LYR subfamilies. The top 10 most conserved residues contain a YNF motif (positions 29–31) with Y27 and N28 particularly strongly conserved, a RQ[TVA] motif (positions 71–73), an LV motif (positions 84–85), an isolated arginine at position 37 and a serine/glycine at position 76 (Fig 6). Among these residues, the YNF motif stood out using various LYR backgrounds, e.g. taking as background not only specific LYR subfamilies, but all LYR family members that were not categorized as orthologs of ISD11 in the OrthoMCL database [36]. This YNF motif is in a non-structured part between helix 1 and 2 in the secondary structure prediction based on the whole LYS family. We thus propose that these residues are involved in the interaction with Nfs1 proteins. Unfortunately, the same analysis was not possible for Nfs1 because, since we have shown that Isd11 binds both Nfs1 and IscS, we could not search for residues specific to eukaryotic orthologs of Nfs1 and not conserved in bacterial orthologs of IscS.

## Discussion

Isd11 is a small protein exclusively present in eukaryotes with no obvious equivalent in prokaryotes. We considered at various stages the possibility that ISD11 could have evolved from YfhJ (or IscX), a component of the ISC operon in bacteria and mostly absent in eukaryotes [42]. We could not, however, find any support for this hypothesis and from the little we know about the functions of these two proteins it seems unlikely. Isd11 is an essential protein in yeast where it takes part in iron-sulfur cluster biogenesis as shown by evidence that depletion of *Isd11* results in impairment of this pathway [20, 21]. In humans, loss of the ISD11 function results in Combined Oxidative Phosphorylation Deficiency 19 (COXPD19), a mitochondrial disorder characterized by respiratory distress, hypotonia, gastroesophageal reflux and lactic acidosis in neonates [7]. This disease was recently associated to a homozygous R68L mutation in the *LYRM4* gene on chromosome 6p25, which encodes the ISD11 protein. Conversely, YfhJ is not essential [43].

As opposed to previous studies, we obtained ISD11 soluble when expressed in bacteria. The tendency of ISD11 to aggregate and be present in a polydisperse population prevented us from carrying out a thorough characterization of the structure by NMR. Attempts to crystallize the isolated protein have also failed so far. We can rationalise the behaviour of the isolated ISD11 as being typical of “complex-orphan proteins”, that is proteins prone to aggregation and/or unfolding if not stabilised by a co-factor or a protein partner [31]. This conclusion agrees with the knowledge that the presence of Isd11 is in most species connected with the presence of Nfs1 and with evidence showing that the role of Isd11 is to stabilize the fold of Nfs1 [20, 21], a protein otherwise also prone to aggregation and degradation [20]. It will be interesting in the future to understand how co-expression of these proteins, which are coded on different chromosomes, is regulated.

While purifying recombinant ISD11 expressed in bacteria, the co-purification of the protein with bacterial IscS came as a surprise. This behaviour does not of course have direct functional significance but is of interest because it suggests a conservation between the IscS and Nfs1 families also at the level of partner recognition. Our findings suggest a possible strategy to obtain a structure of Isd11 through crystallizing the complex with IscS, a protein more easily produced than Nfs1.

Finally, we reasoned that only Isd11 proteins within the more general LYR complex I protein family seem to be implicated in iron-sulfur cluster biogenesis. We thus used this information to predict residues involved in the interaction with Nfs1 proteins. We found three motifs that are more specifically conserved in ISD11 orthologues. Our approach was based on evolutionary considerations and thus very different from the approach adopted in a recent study of the ISD11/NFS1 interactions which used yeast and cell lines as models and looked at the effect of mutations on cell survival [44]. The RQ motif we identified here near the C-terminus agrees well with residues suggested in the previous study as important for recognition (F40, L63, R68, Q69, I72, Y76, L81 and E84). It must however be pointed out that, in the experimental study, the authors did not distinguish between residues directly involved in interaction and those important for protein folding. It is thus well possible that some of the suggested residues are buried in the hydrophobic core and contribute to fold stability rather than to the interaction. More work is now necessary to obtain a high resolution picture of Isd11 in complex with either Nfs1 or, alternatively, IscS. When this is obtained, it will be possible to compare the properties of this difficult and sticky protein when in isolation as described in the present work and in the complex.

## Supporting Information

S1 Table Peptide Table Report for MS analysis. (XLSX)
